# Voltage-Driven All-Solid-State Ionic Control on Co/CoO Antiferromagnet/Ferromagnet Exchange Bias

**DOI:** 10.1021/acsnano.5c03052

**Published:** 2025-05-28

**Authors:** Gabriel Vinicius de Oliveira Silva, Labanya Ghosh, Rabiul Islam, Clodoaldo Irineu Levartoski de Araujo, Guo-Xing Miao

**Affiliations:** † Department of Electrical and Computer Engineering, 8430 University of Waterloo, Waterloo, Ontario N2L 3G1, Canada; ‡ Institute for Quantum Computing, 8430 University of Waterloo, Waterloo, Ontario N2L 3G1, Canada; § Laboratório de Spintrônica e Nanomagnetismo, Departamento de Física, Universidade Federal de Viçosa, Viçosa, Minas Gerais 36570-900, Brazil

**Keywords:** all-solid-state ionic gating, battery-like electronic device, voltage control of magnetism (VCM), magneto-ionics, antiferromagnet/ferromagnet exchange bias tuning, spintronics, iontronics, spiontronics

## Abstract

Spintronics traditionally relies on a large electric current to create magnetic fields or spin torques to manipulate magnetic properties, which inevitably leads to undesirable energy dissipation. Alternatively, the voltage control of magnetism (VCM) promises significantly lower energy costs. In the context of VCM, magneto-ionics distinguishes itself by leveraging voltage-driven ion transport as an energy-efficient approach to control magnetic properties, including magnetization, coercive field, and exchange bias (EB). Herein, we demonstrate that the voltage-driven ionic control of CoO antiferromagnetism allows manipulation of the magnetic properties in exchange-coupled ferromagnetic Co. In a “battery-like” device geometry, a 5 nm Co film is precisely oxidized to realize the Co/CoO heterostructure that is interfaced with a solid-state electrolyte and an anode-like Li ion source. The cathode-like CoO layer reversibly converts back and forth between Co and CoO under gate biases, even after 1000 cycles. This subsequently influences magnetic switching in the exchange-coupled Co layer, which is directly revealed by anisotropic magnetoresistance (AMR) in the Co channel. Our findings demonstrate an efficient method of all-solid-state, voltage-driven, highly reversible ionic control on magnetic channels, offering additional dimensions of control and mass integration capability for spintronic applications.

## Graphical abstract

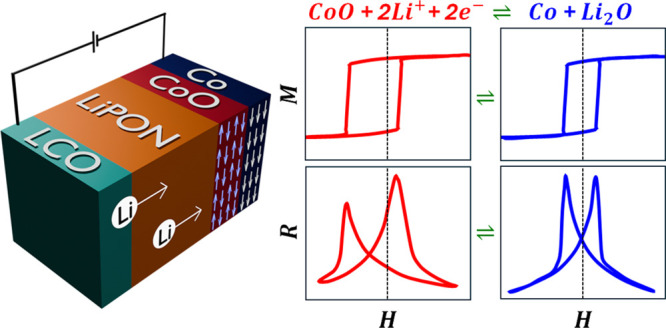

## Introduction

The development of advanced electronic devices has long relied on the use of dielectric materials in traditional metal-oxide-semiconductor field-effect transistors (MOSFETs). This archetypical device has been the backbone of modern electronics,^1,2^ including computers, portable devices, communication networks, among others. Such examples give a glimpse of this technology’s stability, reliability, and scalability. The use of MOSFETs in conventional semiconductors has proven immensely successful,^1,2^ but, from the application perspective, this standard gating method fails when extended to superconductors^3,4^ transition metal (TM) oxides,^5,6^ van der Waals (vdW) 2D materials^7,8^ and magnetic thin films.^9,10^ In a nutshell, standard dielectrics under typical gate voltages fail in inducing sufficient carrier density or triggering phase transition in these emerging materials, resulting in the need for searching for alternative technologies capable of addressing this shortfall at modest gate voltages.

To address this, researchers turned their attention to ionic-liquid-based gating methods that rapidly became a widely used platform for tuning the properties of a diverse range of materials.^11–15^ The nature of the dielectric component in ionic liquid gating is different from that for MOSFETs. Here, the gate medium is composed of mobile ions, which are electrically insulating but ionically conductive. The interface between the electrode and electrolyte forms an electrical double layer (EDL) of about ∼1 nm,^16^ and an ultrahigh electric field builds up, enabling much higher capacitance than that of oxide dielectrics at comparable voltages.^17^ Consequently, the density and polarity of the charges in the double layer can be precisely controlled with gate bias, and massive modulations of the surface charge density, comparable to those in metallic systems, can be achieved.^18,19^ Beyond the electrostatic gating effect, electrochemical gating with guest-species intercalation may also be feasible in electrolyte-electrode systems, depending on the nature of the electrolyte and the properties of the target material.^20^

Despite the unprecedented success of ionic liquids, their use is largely limited to single devices, since their liquid state poses challenges for applying them to integrated electronic systems. An alternative yet similar approach called solid-state electrolyte (SSE) gating has emerged to tackle these issues. Such electrolytes retain the benefits of electrolyte gating while still permitting mass adoption of robust and scalable technologies. From the fundamental research aspects, SSE gating is also advantageous to ionic liquids because it allows back-gate geometries, leaving the top surface accessible for surface-sensitive experimental techniques as well as in situ characterizations.^21^

To use ionic gating for tuning material properties, it is important to outline some key takeaways on how it differs from conventional electric-field (CEF) gating methods. The ionic gating excels in power efficiency since it operates on electrochemical principles, where the required energy is minimal compared to conventional electric-field-based methods. One key advantage is the strength of gating: with chemical doping into the materials (intercalation), the gating effect is much stronger than the typical CEF gating, which is limited by the screening depth and therefore problematic in more conducting systems. Consequently, ionic gating can gate even metals,^22–24^ which is hardly possible for CEF gating. In comparison, CEF approaches often require much larger voltages and therefore much more energy to realize similar gating effects.^25–27^ As for scalability, ionic-liquid type of gating is challenging due to its liquid form, preventing its use in integrated systems. However, our system does not suffer from this problem, as the solid-state form of the electrolyte permits large-scale integration. The main disadvantage of ionic gating is that the switching speed is limited by the ion mobility, much slower than that of electrons. The typical ion migration faces significant migration energy barriers and hysteresis, while the CEF gating relies on the rapid rearrangement of charge carriers or dipoles, enabling a much faster switching speed. However, these slower dynamics can be advantageous in neuromorphic applications,^28,29^ where gradual and history-dependent conductance changes can mimic biological synaptic behavior, just like what happens in ion-activated biological synapses.^30^

In this work, we utilize an all-solid-state ionic gating approach to tune the antiferromagnetic (AFM) cobalt oxide (CoO) film. We adopt a “battery-like” device layout^29,31^ for effective ion intercalation and extraction (Figure 1a). The electron-beam evaporation (e-beam) grown Co film is precisely oxidized to realize the Co/CoO heterostructure, with CoO behaving cathode-like for intaking Li ions. A solid-state electrolyte, lithium phosphorus oxynitride (LiPON), is used for efficient Li ion migration while staying electrically insulating. An anode-like lithium cobalt oxide (LCO) layer was chosen as the Li ion reservoir. With AMR measurements at room temperature and 10 K, we can monitor the EB and coercive field changes inside the Co channel at the application of gate biases. The tuning of these magnetic properties is highly reversible, even after 1000 cycles. Our research focuses on exploring and unlocking new possibilities in the manipulation of ferromagnetic (FM) effective thickness as well as the modulation of AFM ordering, with a view toward advancing spintronics^32^ and spin–orbitronics^33–37^ technologies. Weakening and restoring the FM and AFM strengths on demand can well assist in the desired magnetic manipulations. Specifically, our approach involves precise control of FM thickness and strength, which could enable a seamless transition between athermal and thermodynamically active devices. This transition may be achieved through the manipulation of the superparamagnetic regime,^38^ potentially facilitating innovative applications in spin valves^39^ or degenerate spin systems.^40,41^ Moreover, reversibly manipulating the strengths of AFM interactions could prove highly beneficial for the development of thermally assisted magnetic random-access memory (MRAM) devices,^42^ which are a critical component of next-generation memory technologies. By leveraging these control mechanisms, we aim to push the boundaries of spintronics and spin–orbitronics, offering new pathways for enhancing the performance and functionality of future electronic and memory devices.

**Figure 1:**
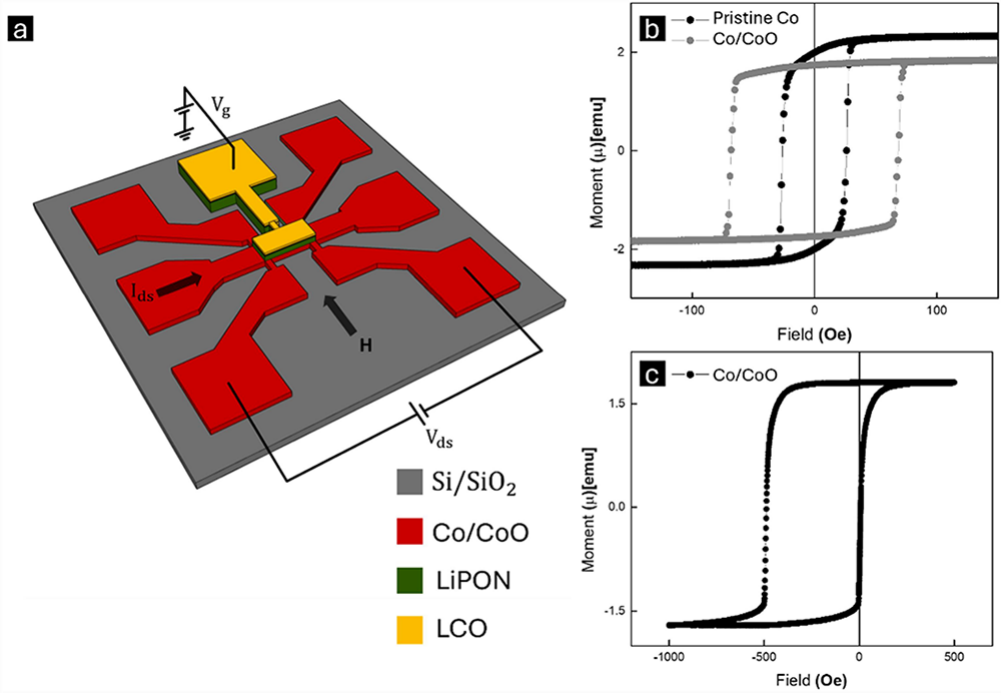
(a) Schematic of the device structure, illustrating a patterned Hall-bar geometry. (b) *M*–*H* measurements of Co and Co/CoO films at 300 K. (c) *M*–*H* measurement of the Co/CoO film at 10 K.

## Results and Discussion

A schematic of the device structure, consisting of a patterned Co/CoO Hall-bar with LiPON/LCO and metallization forming the gate over the active area, is shown in Figure 1a. We fabricated Ti (2 nm)/Co (5 nm) on Si/SiO_2_ substrates and partially oxidized Co to obtain the device stack Ti (2 nm)/Co (5-x nm, partially oxidized by x)/CoO (1.75x nm, expanded after oxidation)/LiPON (50 nm)/LCO (15 nm). The detailed fabrication methods and structural information are described in the “Methods” section. The magnetic behavior of the pristine Co and Co/CoO films at 300 K is investigated using a vibrating sample magnetometer (VSM), as shown in Figure 1b. The pristine Co is subject to room temperature M-H measurement, followed by plasma-assisted oxidation and a second M-H measurement. The oxidation was optimized to maximize the gate efficiency, as too thick CoO films would require larger gate biases to reach full CoO reduction, and the subsequent Li deintercalation becomes challenging and results in a less reversible process. In contrast, too thin of CoO does not generate sufficient EB to influence the channel properties. The extent of oxidation can be tracked with the saturation magnetization changes. For example, the optimum Co oxidation results in a magnetization drop from 2.33 to 1.84 μemu, and a coercivity (*H*_C_) increase from 52 to 136 Oe, as shown in Figure 1c. At 300 K, no EB is observed because CoO has a Neel temperature of 293 K. Upon field-cooling under +4 kOe down to 10 K, a strong EB of about −490 Oe is measured, as shown in Figure 1c. The temperature dependence of EB, determined by the exchange interaction strength (the Neel temperature) and the grain magnetization stability (the blocking temperature), is demonstrated through magneto-transport measurements on the patterned Co/CoO structure, and details are shown in the Supporting Information (SI), Figure S1. The findings show the strongest EB effect at 10 K and its weakening above 100 K, suggesting that the system reaches its blocking temperature, even though still below its Neel temperature.^43^ Before proceeding with the magneto-ionic measurements with gate biases, we carried out field cooling under positive and negative fields to 10 K, as shown in Figure S2. EB clearly changed the polarity under different applied fields. It is important to highlight that the coercivities found in the magneto-ionic transport measurements are noticeably larger than those measured on unpatterned films. This difference can be attributed to the shape anisotropy introduced by the narrow Hall-bar geometry.^44^

Similar to lithium-ion batteries, Lithium (Li) ions can be driven into the CoO lattice by a positive bias voltage, where they act to reduce the transition-metal compounds. Such a process is facilitated by the very low electronegativity of Li^45^ for activating strong reductions along with the smallness of Li ions for being accommodated in the CoO matrix.^46,47^ The overall reaction is given by

$$\mathrm{CoO}+2{\mathrm{Li}}^{+}+2e^{-}↔\mathrm{Co}+{\mathrm{Li}}_{2}O$$

where the injection of lithium ions reduces Co^2+^ into metallic Co^0^. This reaction is highly reversible, and a corresponding increase in magnetization is observed^48^ when it is driven forward, aligned with the conversion of CoO into Co. The ground state of CoO has AFM ordering of type-II,^49,50^ with spins aligned parallel within the (111) plane and antiparallel between adjacent planes. Upon lithiation, the stronger affinity of O with Li releases Co from the O-mediated superexchange and forms the itinerant magnet. This conversion increases the effective Co thickness, therefore the channel conductivity, and at the same time weakens the CoO exchange bias on the channel. The modified magnetic switching properties can be directly probed by AMR measurements on the Co channel.

Figure 2a–h shows the results of the in-plane AMR measurements under different gate biases at 300 K. The Co/CoO heterostructure starts with no EB at room temperature because the Néel temperature of CoO is below 300 K.^51^ Adding Li ions does not lead to AFM ordering, and no EB is observed over any gate bias (*V*_G_). At the lower range of gate biases (0–0.8 V), Figure 2a–d, the *H*_C_, AMR amplitude, and resistance are found to be resilient to changes, indicating that the chemical reduction potential has not been reached. Figure 2e–g, on the other hand, shows more dramatic changes of AMR for *V*_G_ ranging from +1 to +3 V. The gate biases now are large enough to weaken the AFM magnetic anisotropy as more Li ions are injected into CoO, causing a progressive reduction of the CoO layer back to metallic Co. *H*_C_ at *V*_G_ = 0 V is found to be 592 Oe, and gradually reduces as *V*_G_ increases, eventually reaching that of the pristine Co film of 218 Oe at *V*_G_ = +3 V, as shown in Figure S7. Despite the reduction in *H*_C_, the AMR ratio still sees minimal changes. This indicates that although the anisotropy is reduced, the intrinsic spin-dependent scattering mechanisms, mostly spin–orbit interactions in origin, remain largely unaffected. At a large, reversed bias of −3 V, all Li ions are deintercalated from the CoO layer, and this layer is oxidized back to CoO, as shown in Figure 2h. Figure 2a, h shows the same *H*_C_, demonstrating that our method is highly reversible. For a direct comparison of these results, see the combined plot in the SI, Figure S9a. We also investigated the EB behavior in our Co/CoO heterostructure under applied gate biases by carrying out field cooling procedures down to 10K, as shown in Figure 3a–h. Figure S5 summarizes the overall trends for *H*_C_, AMR ratio, EB, and resistance as a function of *V*_G_ at 300 and 10 K.

**Figure 2:**
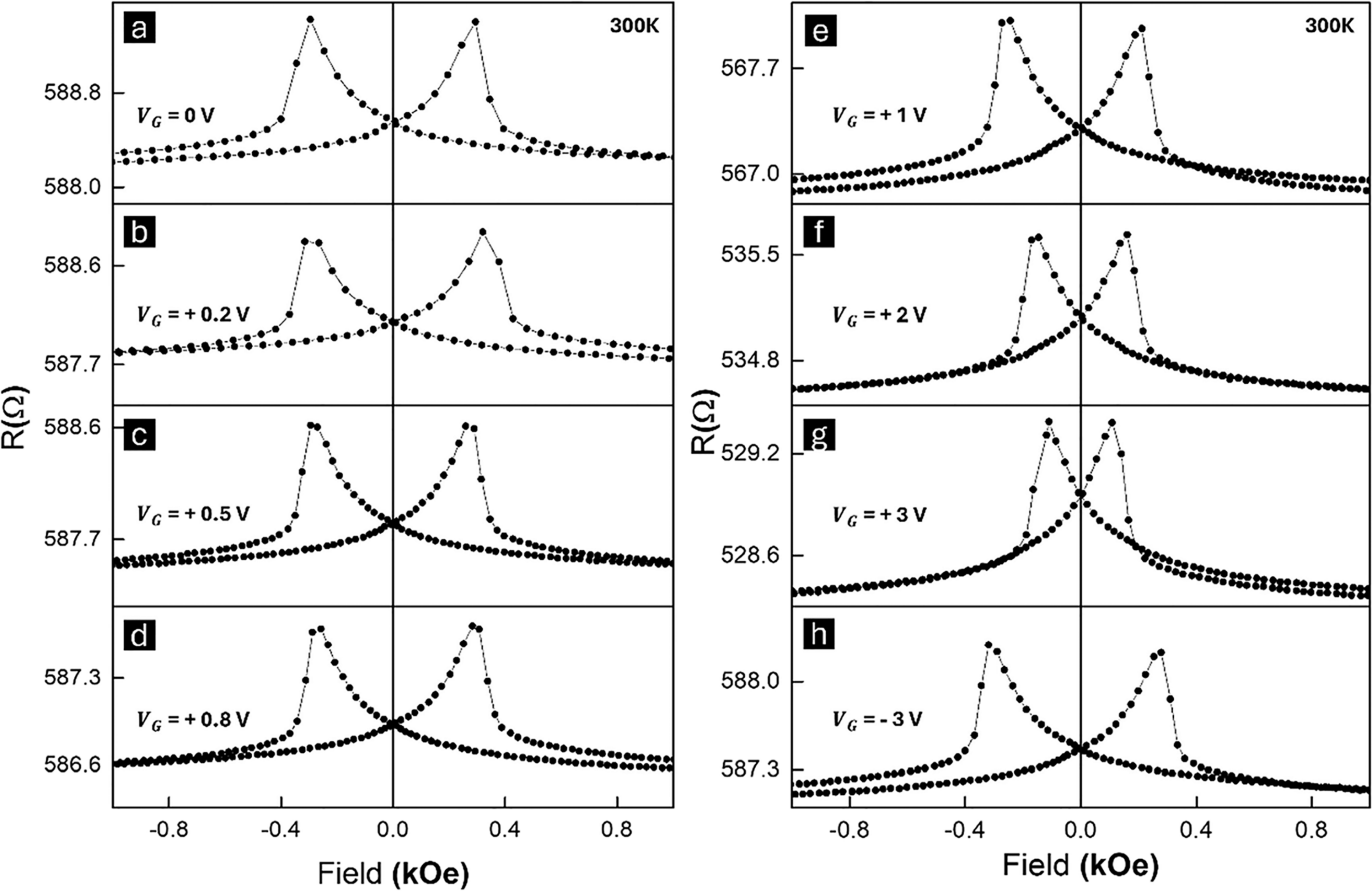
Magneto-ionic transport measurements at 300 K. (a–h) AMR response of Co/CoO Heterostructure at different gate biases (*V*_G_ = 0, +0.2, +0.5, +0.8, +1, +2, +3, and −3 V). The curves were obtained with an in-plane field perpendicular to the current direction.

**Figure 3:**
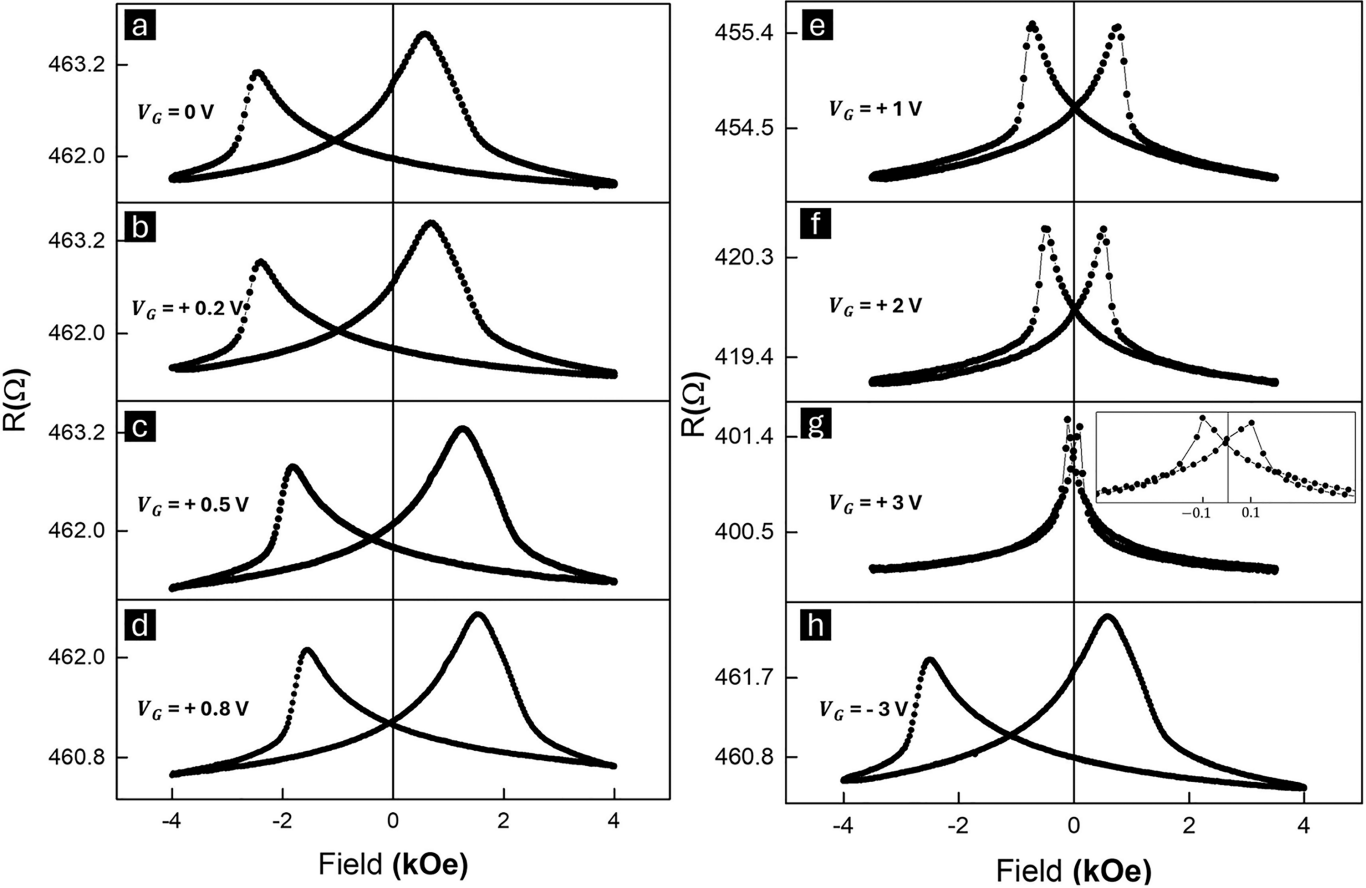
Magneto-ionic transport measurements at 10 K. (a–h) AMR response of Co/CoO Heterostructure at different gate biases (*V*_G_ = 0, +0.2, +0.5, +0.8, +1, +2, +3, and −3 V). The device was field cooled with an in-plane field of +4 kOe perpendicular to the current direction, and the curves were obtained in the same field direction.

As for the low-temperature measurements, two considerations turn out to be important–first, the gate bias is always changed at 300 K, and the sample stays biased for 15 min before proceeding. It has been reported that below 220 K, the ion-induced electrical transport through LiPON becomes negligible,^29^ and that below 140 K, the signal associated with Li motion in LiPON becomes undetectable in Potentiostatic Electrochemical Impedance Spectroscopy (PEIS) and Isothermal Transient Ionic Current (ITIC) measurements.^52^ Therefore, as the temperatures approach cryogenic ranges, ion mobility significantly drops, aligning with the understanding of ion-freezing at low temperatures. We carried out temperature-dependent gate bias scans at different temperatures in our system, as shown in Figure S4, and the ion motion becomes negligible below 200 K. Second, before carrying out field cooling, the sample is heated to 320 K with an in-plane magnetic field of +4 kOe, while the gate bias remains on, to ensure the full development of the desired EB, even though the Néel temperature (*T*_N_) of CoO films with similar thickness is found to be below 300 K.^53,54^ The warming and cooling rates and times are held the same throughout all measurements for consistency. In addition to that, for each field cooling, only the first measurement loop is used, since successive sweeps of H at a given target temperature affect the apparent strength of the EB, a phenomenon known as the training effect.^55^ The training effect is revealed to be more pronounced in Co/CoO systems where the thickness of CoO is ≤5 nm,^56^ which matches our case with the full Co/CoO stack approximately 5 nm thick. Figure S3 shows the training effect in our system upon two consecutive measurements at no gate bias. For consistency, after taking one single AMR measurement at 10 K, the sample is heated back to 300 K, and *V*_G_ is changed for the next measurement. As Figure 3a–d shows, *H*_C_ is significantly increased at 10 K, and it is consistent with the spin-flop coupling mechanism between the Co and CoO spins,^57^ which creates an additional energy barrier that the magnetic system must overcome to reverse its magnetization. Moreover, the exchange bias field (H_EB_) at 10K under no gate bias is found to be 1055 Oe and progressively reduces as *V*_G_ increases until being completely suppressed at *V*_G_ = +1 V. The cause of such a strong modulation even with a very low voltage range can be attributed to the addition of LCO as a Li reservoir,^58^ which added a significantly higher ion flux through LiPON.^59,60^ Interestingly, the lower gate bias range (0–0.8 V) has almost no impact on *H*_C_ that stays around 3 kOe, while *H*_C_ quickly drops to 1486 Oe at *V*_G_ = +1 V and to 212 Oe at *V*_G_ = +3 V, as shown in Figure 3e–g. The modification to material properties–such as electronic,^61,62^ superconducting,^63^ magnetic,^64^ renewable energy,^65^ catalytic,^66^ and optical,^67^ using electrolytic media is widely reported in the literature, but it is rarely reported for solid-state gates. The behavior of *H*_C_ and EB at 10K can be understood by considering the change in the thicknesses of the antiferromagnetic CoO layer coupled to the ferromagnetic Co layer under gate biases. For gate biases in the 0.2–0.8 V range, we assume a minimal to no CoO-to-Co conversion, so the domain wall dynamics remains unaffected, resulting in no change to *H*_C_.

Surprisingly, while *H*_C_ remains nearly unchanged, the EB drops substantially within the same range. In FM/AFM bilayer systems, the interaction at the interface between the ferromagnetic spins and the uncompensated spins of the antiferromagnet is commonly modeled using bilinear (*r*_1_) and quadratic (*r*_2_) coupling terms, where *r*_1_ causes the bidirectional anisotropy and *r*_2_ the biquadratic anisotropy. The former is intimately connected to EB, while the latter drives changes in *H*_C_.^68^ Hence, the low gate bias range (0.2–1 V) primarily tunes the *r*_1_ coupling, destabilizing the AFM ordering, while the *r*_2_ remains unchanged. These findings are consistent with the observation that EB relies on a stable and ordered Co/CoO interfacial exchange coupling,^69^ and here, even a *V*_G_ as low as +0.2 V drives enough Li to quickly disturb the unidirectional anisotropy. Beyond *V*_G_ = +1 V, EB is no longer observed, and our system has ‘permanently’ lost the CoO antiferromagnetic order. However, off-stoichiometric CoO_*x*_ dominates in this range, which prevents the channel resistance from changing much. On the other hand, *H*_C_ is not an intrinsic property, and it is constrained by the thin film grainy structures. At *V*_G_ = +1 V, *H*_C_ undergoes a sudden decrease, marking the onset of an increase in Co thickness and a decrease in CoO thickness, as well as a more continuous Co morphology. Within the upper limit of gate biases (2–3 V), the CoO-to-Co conversion continues. As the effective Co layer grows thicker, domain wall pinning effects weaken, making domain wall motion easier and, therefore, reducing *H*_C_. We can roughly extrapolate the thickness-induced *H*_C_ changes to lower gate biases, and lower thickness leads to larger *H*_C_, as expected. The excess change at low biases can be attributed to the contributions from AFM *r*_2_. The AMR ratio also shows a slight decrease over *V*_G_ between 0–0.8 V, and a sudden drop at *V*_G_ = +1 V, as shown in Figure S5b. This suggests that the presence of AFM can also influence the spin-dependent scattering in the FM systems. Similar to that at 300 K, the Co/CoO magnetic features at 10 K are completely recovered under *V*_G_ = −3 V, as shown in Figure 3h. The combined plot in SI Figure S9b provides a direct visual comparison of the deintercalation. Before performing the Li deintercalation at *V*_G_ = −3 V, we first measured the devices at *V*_G_ = 0 V after the full reduction at *V*_G_ = +3 V, and an intermediate retention was observed (Figure S8). This indicates that at room temperature, some Li ions can self-relax back to the Li reservoir after the gate voltage. In order to evaluate the method’s stability and reversibility, we subject our device to a transistor-like transfer curve for a thousand cycles and then check its magnetic responses. Figure 4a shows modulation of the channel potential (*V*_ds_) of the Co/CoO heterostructure under *V*_G_ sweeping. This particular sweep voltage window is chosen such that the cycles mostly overlap, indicating an optimum voltage window for efficient ion injection and extraction. The sweep rate is set to 50 mV/s, and a constant channel current (*I*_ds_) of 10 uA is applied to monitor *V*_ds_. In our battery-like structure, the total amount of ion flux is negligible, and one cannot perform the standard battery CV measurements. Instead, the channel conductance is very sensitive to ion influx and can be monitored continuously. The remarkable electrochemical stability of our system is demonstrated by the largely overlapped cycles, with a small shift of 0.41% when comparing the second and 1000th cycles’ *V*_G_ = 0 V backward sweeps, as shown in the inset of Figure 4a. This stable long-term operation is attributed to the highly stable solid-state LiPON electrolyte, which prevents the continuous decomposition of the electrolyte and protects the CoO from side reactions, unlike what occurs in liquid electrolytes.^70,71^

**Figure 4:**
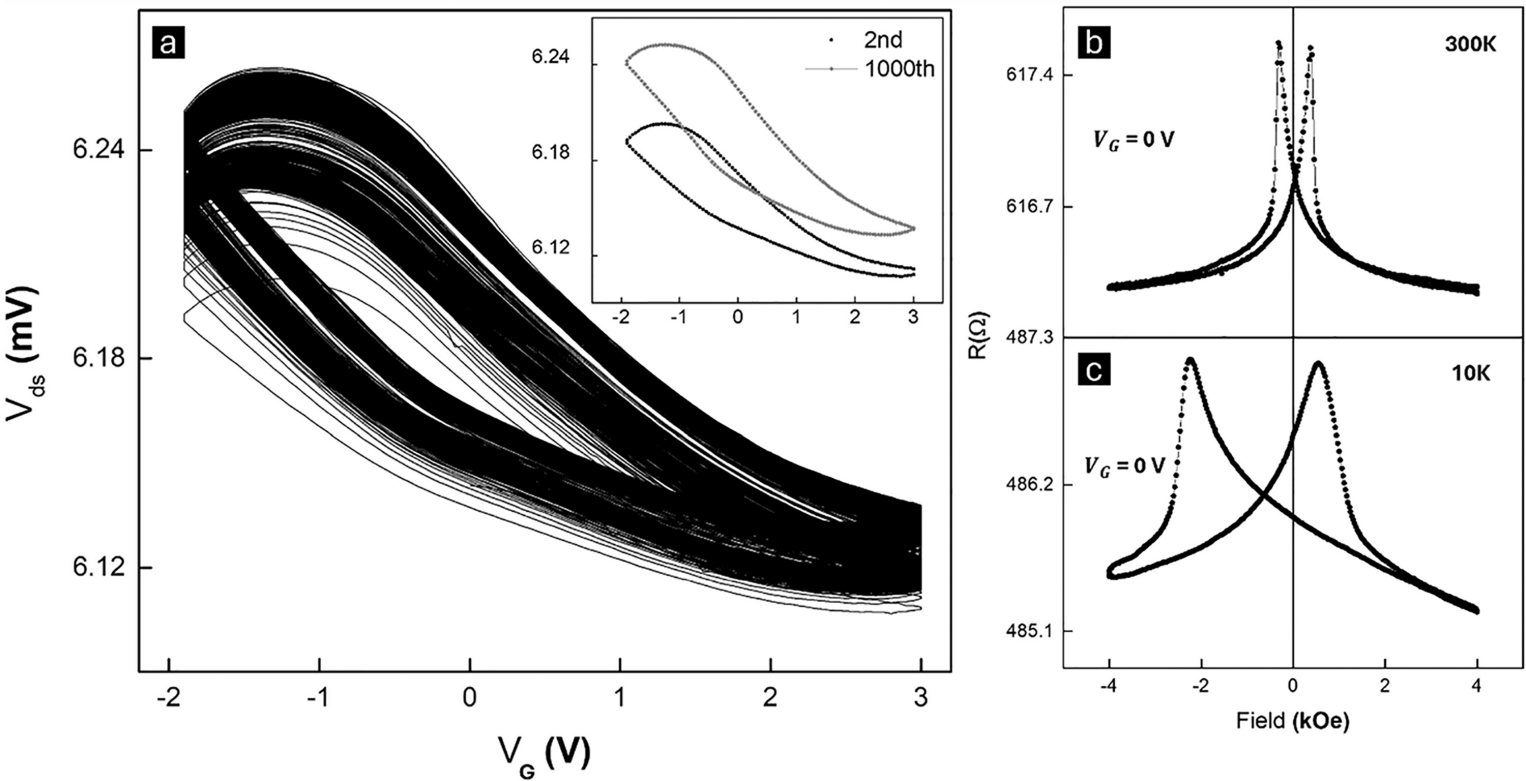
Long-term stability and reversibility evaluation. (a) Li-ion-tuned transfer characteristic curve ranging from −1.9 to +3 V at 50 mV/s, showing 1000 cycles performed at 300 K; AMR response of Co/CoO Heterostructure at *V*_G_ = 0 V after 1000 cycles at (b) 300 K; and (c) 10 K.

Another important aspect of our *V*_ds_–*V*_G_ curve is its hysteretic behavior. The hysteresis loop is a common feature observed in systems that exhibit redox processes, such as in memristive^29,72^ or ion-intercalating systems.^73,74^ Our system involves the intercalation and deintercalation of Li ions, and the observed hysteresis is related to the chemical reaction and ion migration energy barriers during the lithiation (intercalation) and delithiation (deintercalation) processes. Figure 4b,c shows that after 1000 cycles, the magnetic properties of the system are well preserved, and further tuning of the in-plane AMR at 10 and 300 K is shown in Figure S10. We did not directly probe the CoO thickness. Instead, we relied on the VSM-determined saturation magnetization, which points to a CoO thickness likely smaller than ∼2 nm at the starting point. Given that the Thomas-Fermi screening length of Co is about 0.15 nm or approximately one atomic layer,^75^ our Li intercalation did not go beyond it, or we would not be able to fully deintercalate them, and *H*_C_ and EB would not fully recover. Therefore, Li ions can penetrate and react with CoO but not with Co. The Co/CoO interface integrity is critical for EB, while Li intercalation may introduce oxygen vacancies or interfacial reconstruction, especially when dealing with transition metal oxides.^76^ However, such deformations are more pronounced in deep charging cycles, which could induce long-lasting structural degradation. In our study, the applied bias was limited to −3 to +3 V (across 50 nm electrolyte dielectrics), which remains below the critical voltage range (4.5 V^77^) where oxygen loss becomes significant. Despite not having direct evidence, such as interfacial microstructural evolution analysis, we can indirectly probe the interface integrity throughout the device operation by looking at the long-term stability and reversibility, as shown in Figure 4. The shallow “charging” into the ultrathin “cathode” layer can be easily reversed, minimizing structural degradation.

To back up our findings, we performed density functional theory (DFT) calculations, and the results are presented in Figure 5. Initially, we constructed a Co/CoO heterostructure slab to model our system, as shown in Figure 5a (right panel). We performed Bader charge analysis^78^ mainly to quantify the charge transfer and oxidation states of Co atoms due to the presence of Li, which are directly associated with magnetization changes. Figure 5a (left panel) shows the charge density difference color map through a (001) plane cut at the topmost Co layer of the CoO beneath Li. We see that Li donates 0.89e to the surroundings, with the three nearest Co atoms capturing nearly 0.20e each, thereby partially reducing to a lower valence. The charge accumulation and depletion regions are visualized as isosurfaces in Figure 5a (middle panel). The three adjacent Co electron density isosurfaces have different orientations, and thus, the projections on the (001) plane appear different. For clarity, this representation only presents the topmost Co and O atoms at the Li–CoO interface, while the bottom layers are omitted. The Li electron donation influences the local electronic environment of not only Co but also of O. Figure 5b shows the evolution of the magnetization as a function of the Li and O and Li content. The influence of oxygen on magnetization is immediately captured by comparing the total magnetization of the pristine Co slab with that of the Co/CoO heterostructure (details on the total magnetization calculation are provided in the SI, Figure S11). The total magnetization drops from 67.7 to 30.4 μ_B_ upon oxidation, as shown in Figure 5b. With Li incorporation inside CoO, the magnetization gradually recovers, confirming the roles of voltage-driven Li-ion embedment in tuning the magnetic properties of the Co/CoO heterostructure. The incorporation of Li ions into the CoO matrix also disrupts the O-mediated superexchange interactions, leading to a much enhanced magnetization increase per inserted ion. Eventually, when the Li content is large enough, the magnetization will be fully restored, indirectly suggesting the recovery of the other magnetic properties that we measured experimentally.

**Figure 5:**
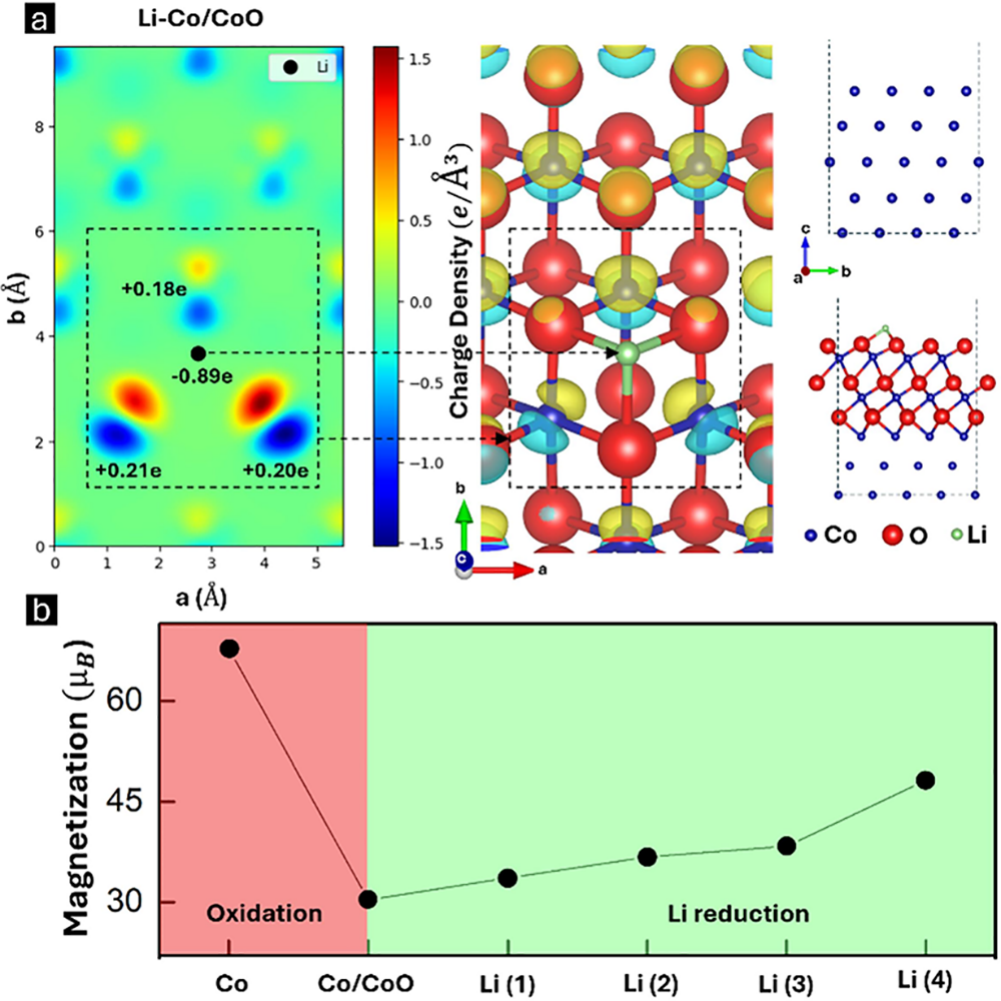
DFT calculations on pristine Co, Co/CoO, and Li–Co/CoO heterostructures. (a) Charge density difference mapped onto the (001) plane at the topmost Co layer beneath Li (left panel), atomic structure of the Li–Co/CoO slab highlighting charge transfer (middle panel), and Co (top) and Li–Co/CoO (bottom) slabs used for Bader charge analysis (right panel). (b) Total magnetization evolution as a function of O and Li contents, illustrating oxidation and Li-induced reduction effects. The slab contains a total of 40 Co atoms.

## Conclusions

In conclusion, antiferromagnetic/ferromagnetic exchange bias, magnetization, and coercivity, tuned by VCM in the framework of magneto-ionics, were investigated in Co/CoO heterostructures. Remarkably, our device can operate reversibly between Co/CoO and Co, even after a long-term operation, proving the robustness and effectiveness of solid-state ionic gating in micro- and nano-integrated devices. Building upon this, we can further extend this method for modifying magnetic switching properties in spintronic applications, facilitating more efficient spin generation, manipulation, and detection in more exotic quantum materials on demand.

## Methods

### Thin-Film Characterization

The electron-beam evaporation (e-beam) grown Co films were first subjected to magnetic characterization using VSM with an in-plane field ranging from −150 to +150 Oe at 300 K. After the plasma-assisted oxidation, we performed another VSM measurement at 300 K with the same parameters, followed by a measurement at 10 K, with a wider field range. Field-cooling transport measurements were performed on patterned Hall-bar geometry, with an in-plane field of ±4 kOe.

### Device Fabrication

The devices were fabricated on Si/SiO_2_ substrates that were ultrasonically cleaned in acetone and IPA. The growth was performed in an AJA sputter/evaporator system with a base vacuum of 2 × 10^–8^ Torr. After loading into the sputter chamber, the substrates were RF back-sputtered for further cleaning of the surface. A Ti adhesion layer was first deposited via DC sputtering, and the samples were subsequently transferred in situ to the evaporation chamber where the Co layer was e-beam deposited at a constant rate of 0.1 Å/s. Next, the Hall bar structure was patterned using a direct-write lithography system (MLA150), and the exposed areas were etched with ion milling using an AJA ion mill system equipped with secondary ion mass spectrometry (SIMS) for end point detection. The patterned Hall bars were then controllably oxidized to form the Co/CoO heterostructures by a downstream plasma oxidation (YES-CV200RFS) at 25 W, 400 mTorr, 175 °C, and an O_2_ flow of 50 sccm for an optimized oxidation time of 200 s (devices with other less-optimized oxidation time had also been fabricated and tested). After oxidation, another lithography step was performed to open the gate electrode area, where the solid-state electrolyte, LiPON, and the Li-reservoir, LiCoO_2_, were deposited by reactive sputtering using a N gas flow of 20 sccm at 35 W, and Ar/O gas flow of 30/3.3 sccm at 75 W, respectively. Subsequently, the gate contact of Ti and Al was sputtered in pure Ar. Finally, one more lithography step was carried out to define the Ti and Al contact pads on the Hall bar electrodes.

### Computational Methods

The first-principles calculations were conducted in the framework of DFT using the Vienna Ab-initio Simulation Package (VASP)^79^ to optimize the pristine Co, CoO structures, and the Co/CoO heterostructure to their ground state and verify the effects of O and Li on magnetic behavior. Initially, the structures of Co and CoO were subjected to a spin-polarized relaxation with a kinetic energy cutoff of 520 eV for the plane wave until the force on each atom is less than 0.01 eV/Å. The total energy convergence criteria is chosen as 10^–5^ eV, and a k-point mesh of 5 × 5 × 5 was employed, with the exchange-correlation functional approximated using Perdew–Burke–Ernzerhof (PBE)^80^ within the generalized gradient approximation (GGA). We constructed slabs for Co (111) and CoO (111) and assembled the heterostructure using VESTA,^81^ averaging the lattice constants and subjecting it to a spin-polarized relaxation with a k-mesh of 5 × 5 × 1. Using the Co/CoO heterostructure, we gradually increased the Li content and monitored the total magnetization, as described in Section 11 of the SI. In addition, we performed Bader charge analysis to compare the charge redistribution in Co/CoO in the presence of Li.

## Supplementary Material



## Notes

Exploration of temperature effects on AMR to refine exchange bias performance (Figure S1), signature of exchange bias in Co/CoO/LiPON at no gate bias (Figure S2), observation of the training effect on Co/CoO/LiPON at no gate bias (Figure S3), clear evidence of ion freezing occurring below 200 K (Figure S4), trends found in *H*_C_ (Oe), AMR (%), exchange field (Oe) and resistance as a function of gate bias (Figure S5), intrinsic effect of LiPON on the Co/CoO system before any gate bias is applied (Figure S6), complete reduction of CoO to metallic Co achieved under a gate bias of +3 V (Figure S7), partial reoxidation of metallic Co observed at zero gate bias following prior reduction (Figure S8), Li-ion deintercalation inducing the transition from metallic Co to CoO under a gate bias of −3 V (Figure S9), demonstration of a stable and tunable AMR effect sustained over 1000 gating cycles (Figure S10), and magnetization analysis based on first-principles DFT calculations (Figure S11) (PDF)
