# Supplementary material for: Voltage-Driven All-Solid-State Ionic Control on Co/CoO Antiferromagnet/Ferromagnet Exchange Bias
Source: ACS Nano. 2025 May 28;19(22):20799–807. doi: 10.1021/acsnano.5c03052 (PMC12164521; doi:10.1021/acsnano.5c03052)
Supplement: Supplementary file 1 [file nn5c03052_si_001.pdf]

Supporting Information

Voltage-Driven All-Solid-State Ionic Control on Co/CoO Antiferromagnet/Ferromagnet Exchange Bias

Gabriel Vinicius de Oliveira Silva[1,2], Labanya Ghosh[1,2], Rabiul Islam[1,2], Clodoaldo Irineu Levartoski de Araujo[3], and Guo-Xing Miao[1,2]*

[1]Department of Electrical and Computer Engineering, University of Waterloo, Waterloo, ON, N2L 3G1, Canada

[2]Institute for Quantum Computing, University of Waterloo, Waterloo, ON, N2L 3G1, Canada

[3]Laboratório de Spintrônica e Nanomagnetismo, Departamento de Física, Universidade Federal de Viçosa, Viçosa, 36570-900, MG, Brazil

E-mail: guo-xing.miao@uwaterloo.ca

1. Temperature-dependent AMR investigation for optimizing exchange bias

The exchange bias is known to have strong temperature dependence[1]. Such dependence comes from thermal instabilities of the AFM coupling in the AFM grains, along with the thermally activated switching of the AFM grains. As expected, we observed EB scaling with decreasing temperatures, as shown in **Figure S1(a)**. The EB becomes very small above 100 K, indicating the system hits its blocking temperature even though still below T_N[2]. Choosing the temperature that gives the strongest EB, i.e., T = 10 K, allows for a more pronounced observation of the gating effect. **Figure S1(b-c)** shows the temperature trend for EB and H_C, consistent with the weakening of AFM domain stability as temperature increases.

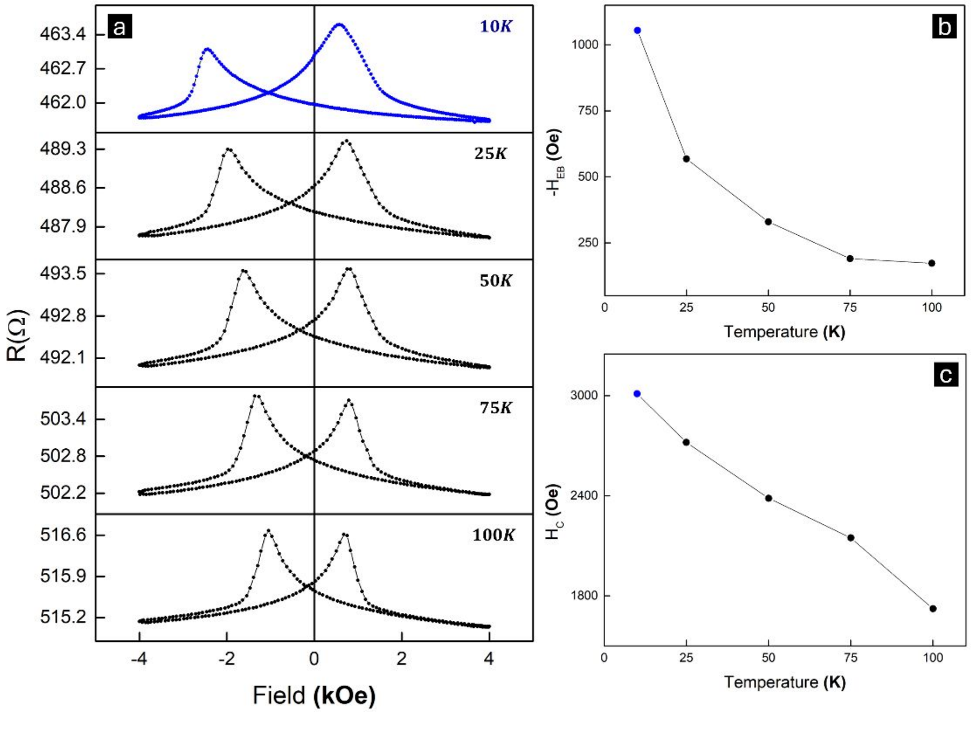

Fig.S1 | (a) AMR curves at different temperatures, showing the evolution of EB. (b) Exchange bias field and (c) Coercivity as a function of temperature, demonstrating their decrease with increasing temperature.

2. Exchange bias fingerprint in Co/CoO/LiPON at V_G = 0 V

The typical hysteresis loop of an AFM – FM system, measured at $T < T_N$ after the field cooling procedure, is shifted along the field axis in the opposite direction to the cooling field[3], as shown in **Figure S2**. This shift in the loop is commonly referred to as exchange bias (H_{EB}). Also, the hysteresis loop exhibits increased coercivity (H_C) following the field cooling procedure – observed here at no gate bias (V_G), and for the measurements under V_G – **Figure 3 (a-h)**, except for **Figure 3(g)**, where CoO has fully reduced back to Co. Performing field cooling for positive and negative fields ensures that what we observe is caused by exchange bias rather than by experimental artifacts or material defects.

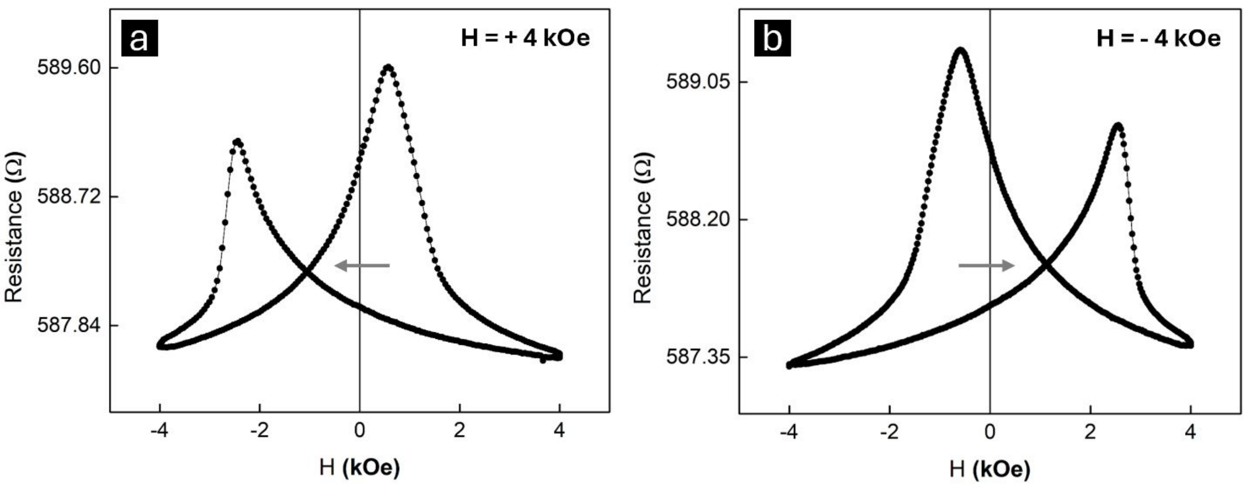

Fig.S2 | (a) Hysteresis loop after positive field cooling at H = +4 KOe, showing a leftward shift with H_{EB}= -1055 Oe. (b) Hysteresis loop after negative field cooling at H = -4 KOe, showing a rightward shift with H_{EB}= +1117 Oe. Both curves were measured at V_G = 0 V.

3. Training effect in Co/CoO/LiPON at $V_G = 0$ V

Another well-known fingerprint of exchange-biased film systems is shown in **Figure S3**. The training effect refers to the gradual change in the exchange bias amplitude over successive sweeps [4,5]. The exchange bias is typically at its maximum in the first sweep after field cooling, and in our system, H_{EB} = -1090 Oe. Upon the second H sweep at 10 K, the system partially reorients the interfacial spins in the AFM layer, and H_{EB} decreases to 545 Oe. It is observed experimentally that $H_E - H_{E\infty} \propto 1/\sqrt{n}$, where n is the number of sweeps. Therefore, the exchange bias gradually approaches its equilibrium value $H_{E\infty}$[6]. This phenomenon is more pronounced in polycrystalline AFM systems[7,8], pointing to the polycrystallinity nature of our CoO.

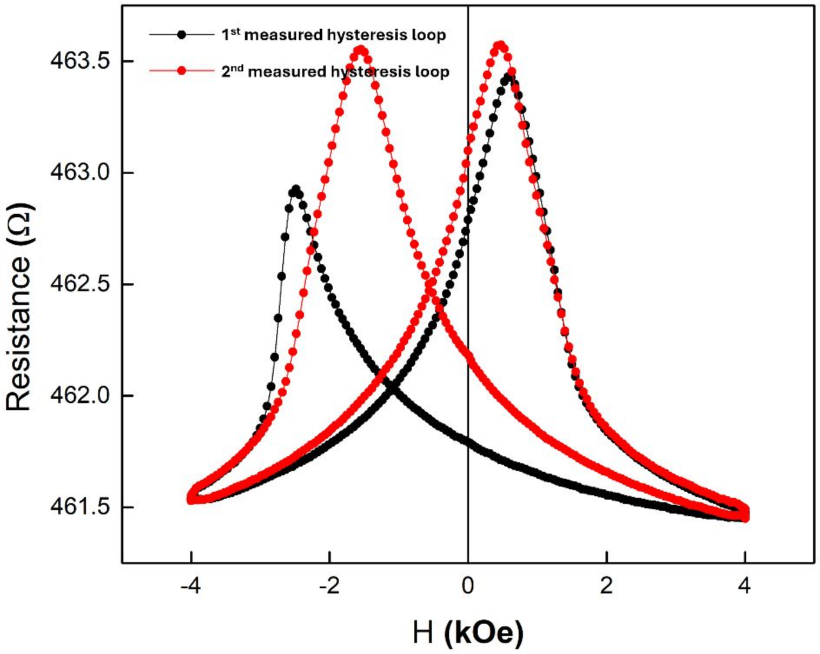

Fig.S3 | In-plane AMR measurements at 10 K demonstrating the training effect: reduction in H_{EB} and H_C upon two consecutive measurements.

4. Temperature-dependent ionic dynamics: evidence of ion freezing below 200 K

In order to give solid evidence of suppressed Li ion motion at cryogenic temperatures, we carried out temperature-dependent gate bias scans, as shown in **Figure S4(a)**. The presence of hysteresis in a gate bias scan is a clear signature of ion motion since it indicates the ion insertion and extraction, with an associated energy barrier. The ion mobility is a strong exponential function with temperature. Our findings show that the ion motion is already very small at 200 K, and practically frozen for even lower temperatures. They are consistent with the literature that below 220 K, Li motion becomes very small[9] (**Figure S4(b)**), and below 140 K, the signal associated with ion motion becomes undetectable[10] (**Figure S4(c)**).

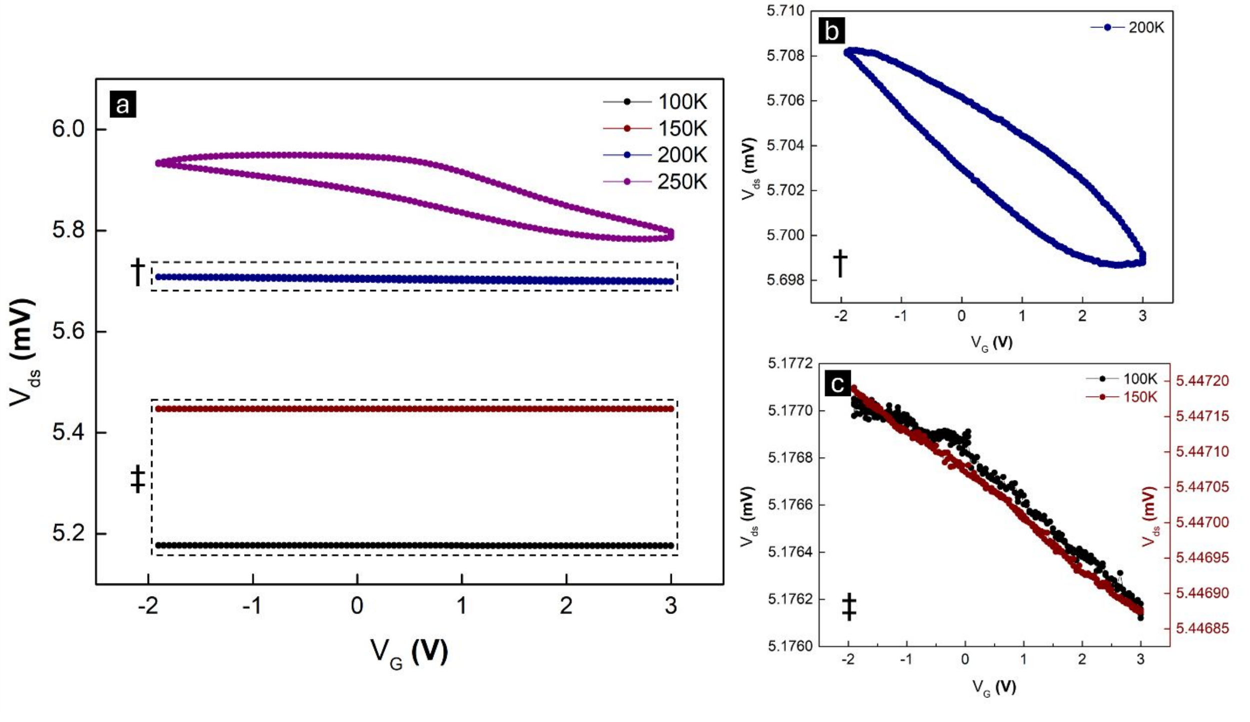

Fig.S4 | (a) Comparison of gate bias scans at different temperatures, showing significantly suppressed ionic activity at lower temperatures, (b) Zoom-in of 100 K and 150 K scans, confirming ion freezing at these temperatures, (c) Zoom-in of 200 K scan, revealing a small but noticeable hysteresis loop, indicating limited ionic motion.

5. Hc (Oe), AMR (%), Exchange field (Oe), and R(Ω) vs V_G

Figure S5 provides a summary of the results presented in **Figure 2** and **Figure 3**. Here, we highlight the trends in H_C, AMR ratio, exchange bias field and resistance as a function of V_G.

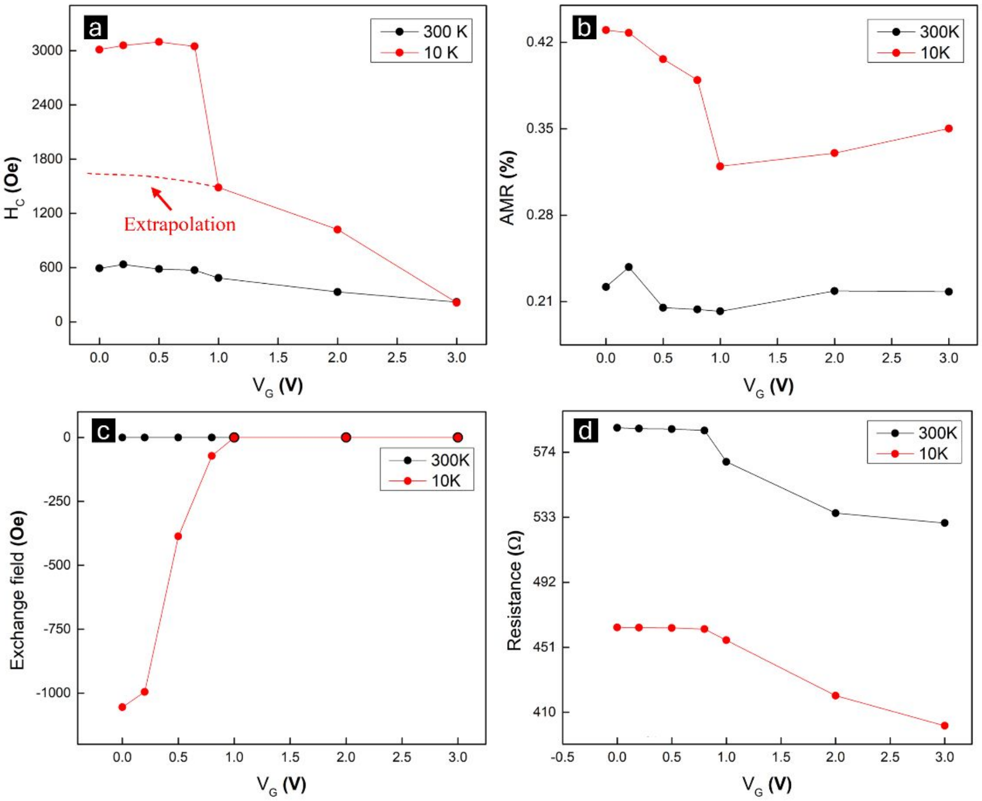

Fig.S5 | (a) Coercive field (Hc, Oe), (b) Anisotropic magnetoresistance ratio (AMR, %), (c) Exchange field (Oe), and (d) Resistance (Ohms) as a function of V_G at 10 K and 300 K.

6. Effect of LiPON itself on Co/CoO prior to applying gate bias

The Co/CoO heterostructure without LiPON serves as a reference to understand the intrinsic magnetic and electrical properties of pristine Co/CoO. Measuring the Co/CoO/LiPON stack prior to applying any gate bias and comparing it to pristine Co/CoO allows us to identify the LiPON contribution. Essentially, the difference between the two systems directly reveals the effect of LiPON itself, as shown in **Figure S6**. As we can see, the simple presence of LiPON already slightly alters the magnetic and electrical properties of the Co/CoO heterostructure. The changes observed are not significant but still noticeable. This is likely due to some Li ions that already enter the CoO matrix during the sputter deposition and reduce it slightly.

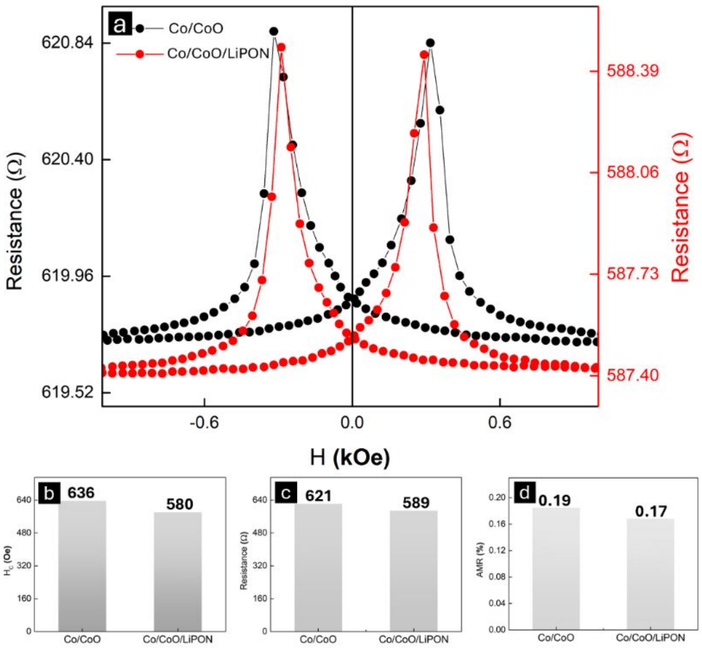

Fig.S6 | (a) In-plane anisotropic magnetoresistance (AMR) with H ⊥ I at 300 K for Co/CoO (black) and Co/CoO/LiPON (red). (b) Coercive field (Hc) for Co/CoO and Co/CoO/LiPON. (c) Resistance for Co/CoO and Co/CoO/LiPON. (d) AMR for Co/CoO and Co/CoO/LiPON. All measurements are performed at zero gate bias (V_G = 0 V) to isolate the effect of LiPON on the Co/CoO heterostructure.

7. Complete reduction of CoO to Co under V_G = +3 V

Here we see the effect of Li^+ ions in fully reducing CoO back to Co under V_G = +3 V, despite the difference in electrical resistance, as shown in **Figure S7**. This is clearly demonstrated by the magnetic signatures – H_C and exchange bias. **Figure S7a** shows an H_C of about 214 Oe for Pristine Co, while 218 Oe for Co/CoO under +3V, both at 300 K. At 10 K, **Figure S7b**, an H_C of 212 Oe is found for CoO under +3V. Furthermore, no exchange bias is observed at this gate bias, as expected for pristine Co. As for resistance, it has partially recovered since Pristine Co presents an electrical resistance of about 442 Ω, the Co/CoO heterostructure a resistance of about 589 Ω, whereas Co/CoO under V_G = +3 V falls in between these states at 529 Ω. The higher resistance in fully reduced Co/CoO is an indication of its modified crystallinity from the pristine Co, even after full reduction.

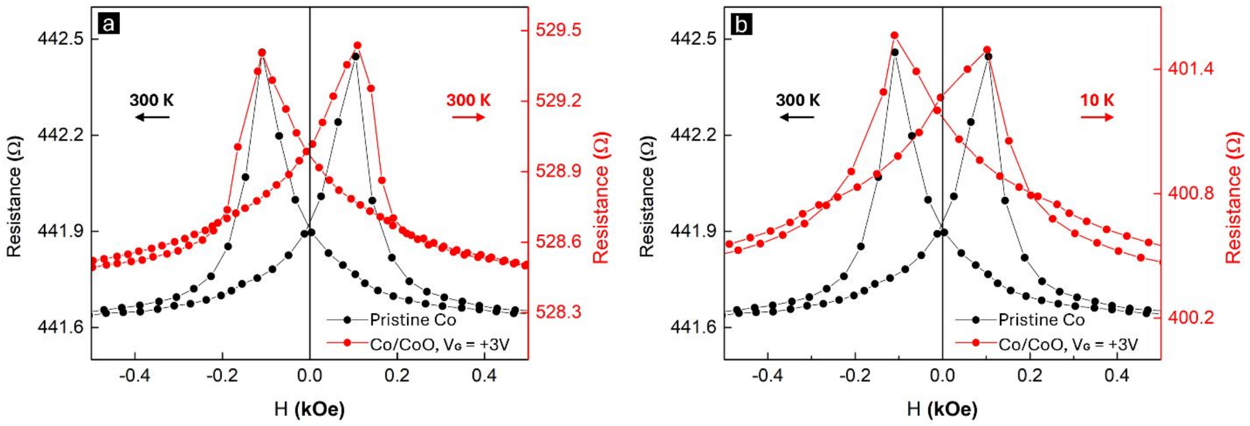

Fig.S7 | In-plane AMR measurements: (a) Comparison of pristine Co (black) and Co/CoO/LiPON (red) at 300 K with V_G= +3 V. (b) Pristine Co (black) at 300 K and Co/CoO/LiPON (red) at 10 K with V_G = +3 V.

8. Partial reoxidation of Co at $V_G = 0$ V post-reduction

We performed an in-plane AMR measurement at $V_G = 0$ V immediately after the full reduction of CoO to Co at $V_G = +3$ V to distinguish between volatile and non-volatile behaviors. **Figure S8** reveals the state of our Co/CoO when V_G is turned off shortly after the full CoO reduction at $V_G = +3$ V. At 300 K, H_C and resistance are found to be 515 Oe and 577 Ω, respectively, falling between the values measured at $V_G = 0.8$ V and +1 V, as shown in **Figure S8a**. Similarly, the same trend is found at 10 K, with $H_C = 2709$ Oe and R = 459 Ω, as shown in **Figure S8b**. Once V_G is turned off, our system is neither at fully reduced CoO ($V_G = +3$ V, permanent retention) nor fully oxidized Co ($V_G = -3$ V, instantaneous reversion), demonstrating a state of intermediate retention, i.e., some ions spontaneously relax back to the ion reservoirs.

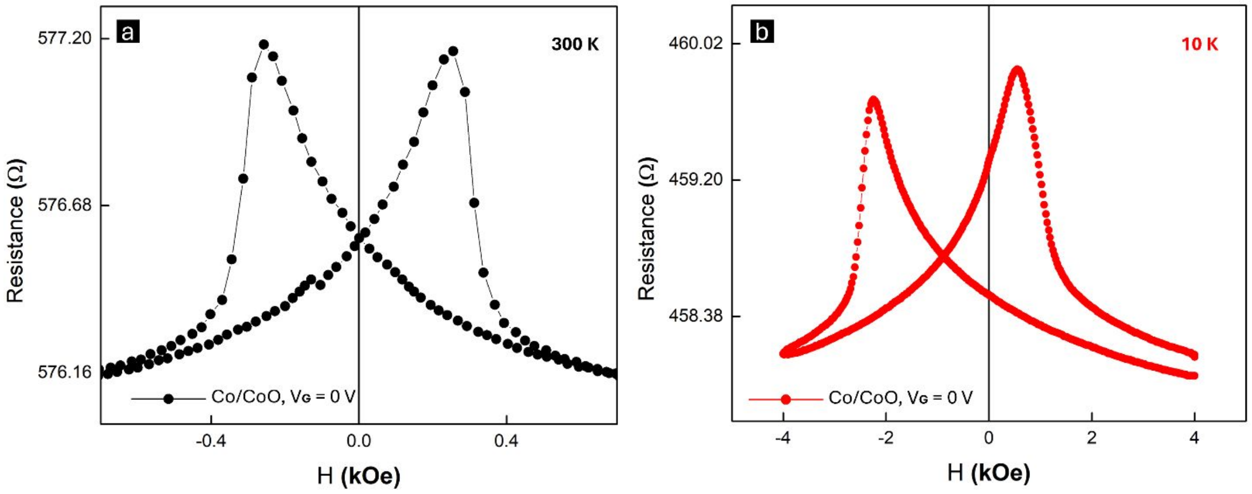

Fig.S8 | (a, b) In-plane AMR measurements at 300 K (a) and 10 K (b) with $V_G = 0$ V after full reduction of Co/CoO to Co at $V_G = +3$ V.

9. Li-ion deintercalation and Co to CoO transition under V_G = -3 V

In order to restore the original Co/CoO heterostructure, we performed in-plane AMR measurements at both 300 K and 10 K under V_G = -3 V. **Figure S9** compares the state of the Co/CoO at V_G = 0 V, before applying any gate bias, with the state at V_G = -3 V after undergoing the full reduction and intermediate retention, as discussed in the previous sections. At V_G = 0 V, H_C = 588 Oe and it is found to be 597 Oe at V_G = -3 V, as shown in **Figure S9a**. Similarly, at 10 K, H_C = 3012 Oe and H_C = 3076 Oe under V_G = 0 V and V_G = -3 V, respectively. Furthermore, H_{EB} = -1188 Oe is found at V_G = -3 V, while it is H_{EB} = -1131 Oe at V_G = 0 V, as shown in **Figure S9b**. The sample resistance is also found to be comparable at both temperatures. In essence, the system returns to its original state under V_G = -3 V meaning it is effective in deintercalating most of the Li^+ ions if not all.

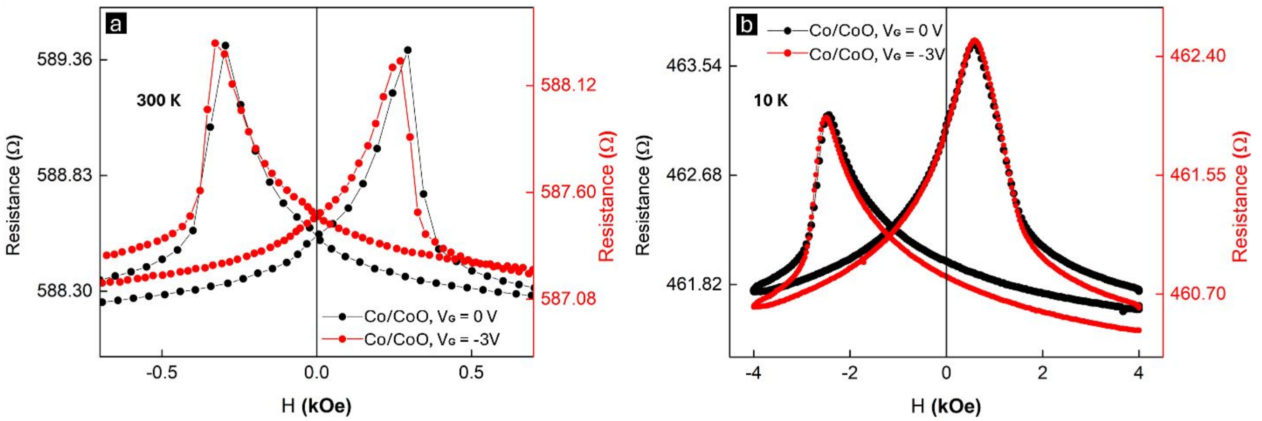

Fig.S9 | (a, b) In-plane AMR measurements at 300 K (a) and 10 K (b) at V_G = 0 V (black) and V_G = -3 V (red), showing the complete transition of Co to CoO due to Li deintercalation.

10. Tunable AMR effect demonstrated after 1000 cycles

After sweeping 1000 cycles and measuring in-plane AMR at $V_G = 0$ V, as shown in **Figure 4b** and **4c**, we then verified the tunability of our devices at the gate bias limits $V_G = +3$ V (CoO full reduction) and $V_G = -3$ V (Co full oxidation). **Figure S10** shows the corresponding in-plane AMR measurements at 300 K and 10 K. The key quantities measured are summarized in **Table S1**. Remarkably, our device demonstrates robust tunability and stability that go beyond 1000 cycles.

Table 1 Summary of device properties at 300 K and 10 K, after 1000 cycles.

	**H_C (Oe)**	**Resistance (Ω)**	**H_E (Oe)**	**AMR (%)**
**300 K** **($V_G = +3$ V | $V_G = -3$ V)**	217 | 595	527 | 590	0 | 0	0.18 | 0.17
**10 K** **($V_G = +3$ V | $V_G = -3$ V)**	214 | 3050	403 | 469	0 | 1025	0.22 | 0.36

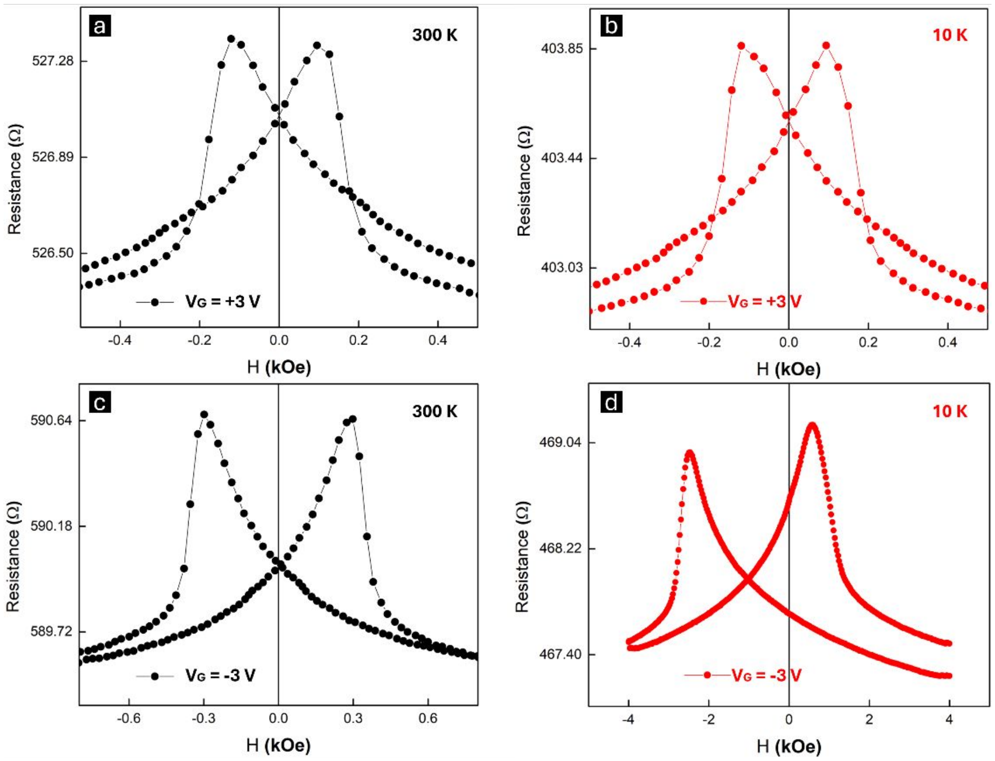

Fig.S10 | (a–d) In-plane AMR measurements at 300 K (a, c) and 10 K (b, d) with V_G = +3 V (a, b) and V_G = −3 V (c, d), demonstrating that after 1000 cycles, the system retains its electrical and magnetic behavior as well as its tunability.

11. Magnetization through First-Principles DFT

Figure S11 shows the DOS comparison between pristine Co, Co/CoO, and Li-doped Co/CoO. Each point in **Figure 5(b)** was obtained by the procedure described here. The Li-doped Co/CoO system shown is the one with the highest Li content we calculated. The total magnetization is given by:

$$M = \mu_B \int_{-\infty}^{E_F} [D_{\uparrow}(E) - D_{\downarrow}(E)]\, dE$$

where $D_{\uparrow}(E)$ and $D_{\downarrow}(E)$ are the spin-up and spin-down DOS, respectively. This integral computes the net difference between the spin states across all energies where the DOS is non-zero.

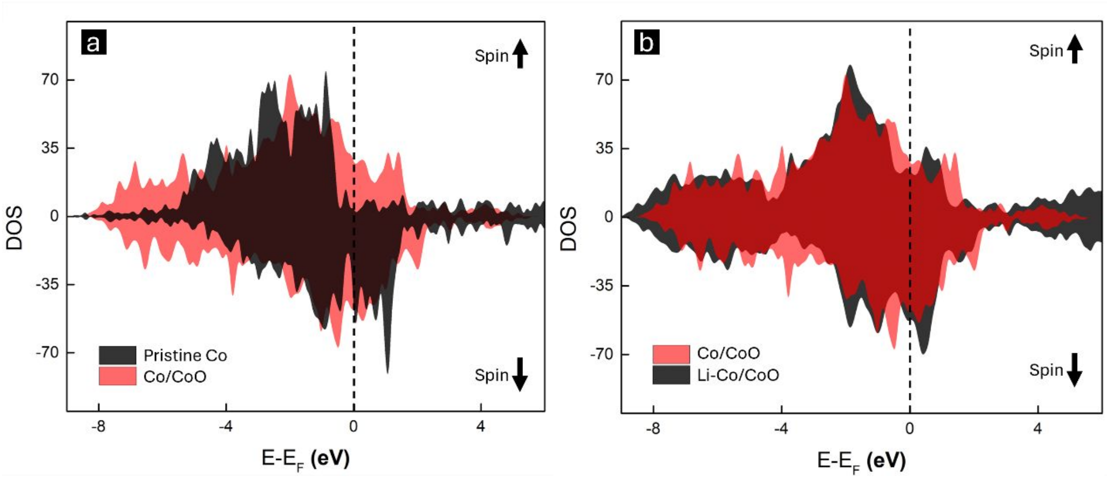

Fig.S11 | Spin-polarized density of states (DOS) for Co systems. (a) DOS comparison between pristine Co (black) and Co/CoO (red). (b) DOS comparison between Co/CoO (red) and Li-doped Co/CoO (black). Spin-up and spin-down states are shown above and below the horizontal axis, respectively. The dashed line represents the Fermi level $(E - E_F = 0\ eV)$.

References

1. Stiles, M. D. & McMichael, R. D. Temperature dependence of exchange bias in polycrystalline ferromagnet-antiferromagnet bilayers. *Phys Rev B* **60**, 12950 (1999).
2. Demirci, E. *et al.* Thickness and temperature dependence of exchange bias in Co/CoO bilayers. *J Supercond Nov Magn* **25**, 2591–2595 (2012).
3. Nogués, J. & Schuller, I. K. Exchange bias. *J Magn Magn Mater* **192**, 203–232 (1999).
4. Tsang, C. & Lee, K. Temperature dependence of unidirectional anisotropy effects in the Permalloy-FeMn systems. *J Appl Phys* **53**, 2605–2607 (1982).
5. Schlenker, C., Parkin, S. S. P., Scott, J. C. & Howard, K. Magnetic disorder in the exchange bias bilayered FeNi-FeMn system. *J Magn Magn Mater* **54**, 801–802 (1986).
6. Schlenker, C. & Paccard, D. Couplages ferromagnétiques-antiferromagnétiques: étude des contractions de cycles d'hystérésis à l'aide d'un traceur de cycle très basses fréquences. *Journal de Physique* **28**, 611–616 (1967).
7. Nogués, J., Lederman, D., Moran, T. J., Schuller, I. K. & Rao, K. V. Large exchange bias and its connection to interface structure in FeF2–Fe bilayers. *Appl Phys Lett* **68**, 3186–3188 (1996).
8. Moran, T. J., Gallego, J. M. & Schuller, I. K. Increased exchange anisotropy due to disorder at permalloy/CoO interfaces. *J Appl Phys* **78**, 1887–1891 (1995).
9. Islam, R., Shi, Y., de Oliveira Silva, G. V., Sachdev, M. & Miao, G.-X. Volatile and Nonvolatile Programmable Iontronic Memristor with Lithium Imbued TiO x for Neuromorphic Computing Applications. *ACS Nano* **18**, 22045–22054 (2024).
10. Le Van-Jodin, L., Ducroquet, F., Sabary, F. & Chevalier, I. Dielectric properties, conductivity and Li+ ion motion in LiPON thin films. *Solid State Ion* **253**, 151–156 (2013).
